# A customizable, low-power, wireless, embedded sensing platform for resistive nanoscale sensors

**DOI:** 10.1038/s41378-021-00343-1

**Published:** 2022-01-14

**Authors:** Stefan Nedelcu, Kishan Thodkar, Christofer Hierold

**Affiliations:** Micro- and Nanosystems, Department of Mechanical and Process Engineering, ETH Zurich, Tannenstrasse 3, 8092 Zurich, Switzerland

**Keywords:** Nanosensors, Electrical and electronic engineering, Carbon nanotubes and fullerenes

## Abstract

Customizable, portable, battery-operated, wireless platforms for interfacing high-sensitivity nanoscale sensors are a means to improve spatiotemporal measurement coverage of physical parameters. Such a platform can enable the expansion of IoT for environmental and lifestyle applications. Here we report a platform capable of acquiring currents ranging from 1.5 nA to 7.2 µA full-scale with 20-bit resolution and variable sampling rates of up to 3.125 kSPS. In addition, it features a bipolar voltage programmable in the range of −10 V to +5 V with a 3.65 mV resolution. A Finite State Machine steers the system by executing a set of embedded functions. The FSM allows for dynamic, customized adjustments of the nanosensor bias, including elevated bias schemes for self-heating, measurement range, bandwidth, sampling rate, and measurement time intervals. Furthermore, it enables data logging on external memory (SD card) and data transmission over a Bluetooth low energy connection. The average power consumption of the platform is 64.5 mW for a measurement protocol of three samples per second, including a BLE advertisement of a 0 dBm transmission power. A state-of-the-art (SoA) application of the platform performance using a CNT nanosensor, exposed to NO_2_ gas concentrations from 200 ppb down to 1 ppb, has been demonstrated. Although sensor signals are measured for NO_2_ concentrations of 1 ppb, the 3*σ* limit of detection (LOD) of 23 ppb is determined (1*σ*: 7 ppb) in slope detection mode, including the sensor signal variations in repeated measurements. The platform’s wide current range and high versatility make it suitable for signal acquisition from resistive nanosensors such as silicon nanowires, carbon nanotubes, graphene, and other 2D materials. Along with its overall low power consumption, the proposed platform is highly suitable for various sensing applications within the context of IoT.

## Introduction

Recent studies have shown that poor air quality is a significant cause of premature death. WHO estimates worldwide casualties of seven million per year^1^. Conventional air pollution monitoring solutions are based on gas chromatography, which leads to relatively large, heavy, and expensive equipment. In addition, such equipment is stationary; requires high installation cost and strict maintenance routines. This has led to increased demand for portable, low-power consuming, customizable gas sensing platforms^2^.

Air quality monitoring systems are used in heating, ventilation, air conditioning systems, air purifiers, and IoT applications. Various IoT applications were developed during the last decade for sensing physical events and transmitting sensor data via wireless communications^2,3^. For example, modern, portable, IoT compatible solutions^4–6^ have enabled air pollution monitoring on a larger scale with the potential for very high spatiotemporal coverage at only a fraction of the cost^7^. Such sensor technology facilitates expanded use by communities, enabling new applications and increasing data volume and access^8^. To compare the results with other available sensors for ambient gas monitoring^9^, we will refer to the regulatory requirements and exposure limits for NO_2_. According to the EU ambient air quality limit values set by directive 2008/50/EC for the protection of human health^10^, the maximum admissible NO_2_ hourly limit value for urban areas is set to 140 μg/m^3^ (corresponding to around 72 ppb) (*Assuming an ambient pressure of 1* *atm., µg/m*^*3*^ = *(ppb) ·(12.187) ·(M)/(273.15* + *°C) where M* = *46* *g/mol represents the molecular weight of NO*_*2*_.), whereas on a yearly average, the NO_2_ level shall not exceed 40 µg/m^3^ (corresponding to around 21 ppb). An example of field NO_2_ daily average result in Europe is presented in Fig. S1. The United States Environmental Protection Agency (EPA), sets the hourly limit standard to 100 ppb and the annual average to 53 ppb. Recently, a large number of commercial sensors^11–13^ can accommodate measurement intervals recommended by the EU, e.g., MAK^14^ concentration range for NO, NH_3_, CO, CO_2_, NO_2_, or O_3_. A comprehensive review of available sensors for ambient gas monitoring can be found in^9^. Energy efficiency, size, and weight are among the most critical design parameters of an embedded sensor platform with System-on-Chip integration^15^. The commercial sensing solution presented in^11^ proposes a similar portable system on a PCB (60 mm × 75 mm) (see Table 1). It is based on commercial off-the-shelf sensors offering multiple gas (O_3_, NO_2_, CO, NH_3_, VOC, H_2_S, SO_2_, and CH_4_) sensing capabilities. Despite the broad range of gases, it requires voltages above 11 up to 24 V with a total power consumption of 2.5–6 W. The configurability of the system is performed using hardware switches. The output resolution is limited to 8 bits without local storage capabilities or wireless data transfer. Another sensing solution is presented in ref. ^12^, offering a compact CO_2_ module (30 mm × 15.6 mm × 8.6 mm) for indoor air quality monitoring. It is a single gas sensor operated at 5 V, drawing 20 mA up to 200 mA of current. The signal is updated every 5 s and it features a proprietary self-calibration algorithm. However, this system is not reprogrammable and does not offer an embedded wireless transmission. For data transfer, an I2C standard interface is available. More recent work is presented in^13^ and proposes a personal wearable multi-pollutant monitoring platform based on commercial off-the-shelf gas sensors. This solution tackles the challenge of low-cost MOX sensor calibration with the help of neural networks for updating the parameters with minimal user intervention. The system integrates two sensors for O_3_ and CO_2,_ drawing 50 mA when both sensors are operated. The system demonstrates accurate measurement results in the presence of human interferences. Another work^4^ proposes a similar monitoring system for CO, SO_2_, and NO_2_ temperature and pressure based on commercial sensors. The system offers 16 bits of resolution and reprogrammable software with the help of a µC. It is powered by a 3.7 V Li-Poly battery cell, consuming an average power of 150 mW including the BLE connection. The capability of reprogramming the platform is however not explored. Although it relies on embedded software, it does not use the full capabilities of building custom readout functions or involving sensor signal calibration procedures. All of the aforementioned solutions are using non-SMD or bulky electrochemical sensing elements.

**Table 1:** Summary of performance comparison

Specification						
		This Work	^11^	^12^	^13^	^4^
System	Supply [V]	5	11	5	3.6	5
	Connectivity	BLE/serial	I2C /serial	I2C/UART	BLE	BLE
	Sensor material	CNT	MOS	NDIR for CO_2_	MOX for O_3_ and CO_2_	Electrochemical for NO_2_
	Range [ppm]	0–0.2	0-0.2	0–50,000	0–0.2	0.01–50
	Response time [min.]	12	< 1	< 3	< 1	<1.2
	Resolution [ppm]	0.023^a^/0.052^b^ (3*σ* LOD)	0.001 (LDL)	+ /−30 ± 3%ss	O_3_:0.004;CO_2_:64ss	±0.02
	Temp./R.H. [°C/%]	22 °C 0% R.H	0 to 50 °C 5 to 95% R.H.	−10 to 60 °C 0 to 95% R.H.	−40 to 85 °C 10 to 95% R.H.	−30 to 60 °C 15 to 85% R.H.
	Area [cm^2^]	9.5 × 6.5	6 × 7.5	0.3 × 1.5	–	–
	Power [mW]	Avg. 60 @ 3 SPS	2500–6000	Avg. 125	Avg. 180	175
			^19^	^25^	^17^	^22^
Sensor(s)	Detector type	CNT	Graphene	In_2_O_3_-rGO	SnO_2_-NW	Ag-S-rGO
	Limit of detection [ppb]	23 (3*σ*)^a^	100	50	100	500
	Detection principle	Resistive	Hall bar	FET	resistive	FET
	Power [µW]	<0.025–0.5^c^	–	–	20	–

^a^For SD mode

^b^For QSS mode

^c^In QSS mode @ 1 ppb and SH mode

Nanomaterials^16^ such as nanowires^17,18^, graphene^19–21^, modified graphene^22,23^, graphene composite^24,25^, carbon nanotubes (CNTs)^26–28^, and metal oxide (MOx) nanocomposite structures^29–31^ have been the subject of extensive research for sensing applications^32^ due to their low dimension and high surface-to-volume ratio. A complete H_2_S sensing system based on SnO_2_ nanowires and dedicated front-end electronics, data post-processing, and storage^33^. A NO_2_ gas sensor based on SWCNTs as a MEMS structure has been demonstrated in ref. ^34^ with a detection trace level from 1 to 5 ppm. Although the sensor resistance exhibits linearity on exposure to NO_2_ gas concentrations from 1 ppm to 5 ppm, the detection range is higher than the EU limit of 21 parts per billion (ppb) with an averaging period of 1 year^10^. Numerous technological challenges of nanomaterial transducers, such as device variation^35^ and ON current decrease over time as reported in ref. ^36^, remain unknown.

This work proposes a versatile embedded system that facilitates interfacing of such nanosensors using software configurable front-end readout electronics. The system demonstrates an SoA interface to ultra-sensitive CNT nanosensors for gas sensing applications, operable within the MAK limits required by the EU standards.

## Embedded hardware

**Fig. 1:**
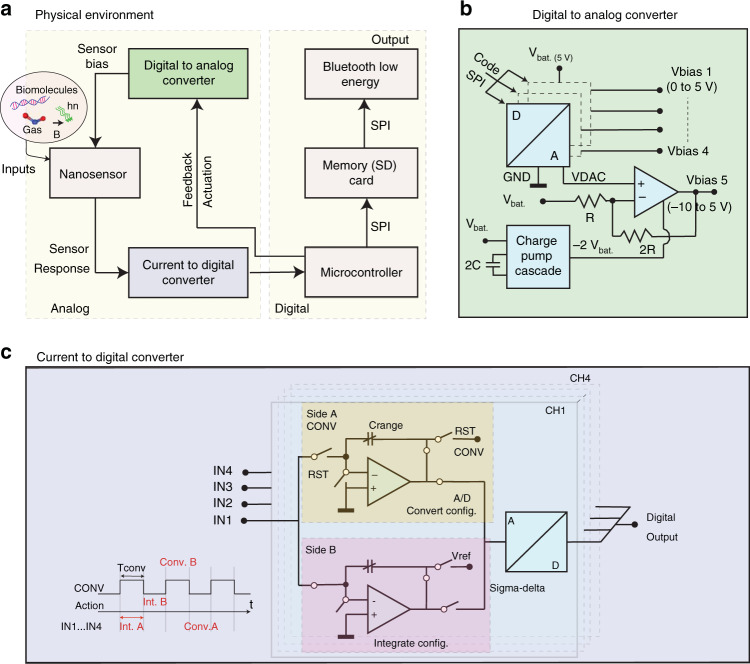
**a** Schematic of the embedded platform divided into two parts: the analog section including nanosensor within a control loop with DAC actuation and sensor response fed to a CDC. The digital section comprises the microcontroller, SD card, and BLE peripherals connected using an SPI. **b** DAC actuation bias block composed of three dual-channel DACs sourcing [0…+5] V on Vbias1…4 and a two-stage inverting charge pump for rescaling the unipolar [0…+5] V range into a bipolar [−10…+5] V range on Vbias5 with the help of an op-amp and two resistors namely R, 2R. **c** CDC adapted from ref. ^50^, illustrating four input channels, each connected to two discrete-time charge integrators operated alternatively as depicted in the timing diagram

At the core of the platform design, the ATmega2560 microcontroller (µC) is used, which features flexible timer/counters for external interruptions, a serial peripheral interface (SPI) including a serial port, and software-predefined power-saving modes. The µC offers short start-up times and low power consumption (~3 mW at 1 MHz in Active Mode)^37^. The data management is ensured by a local SD card storage connected via the SPI interface. An additional transmission (TX) module was chosen to support the wireless transmission. Most IoT solutions are based on Wi-Fi communication featuring different data protocols^38^, with a few hundred meters of link budget, 16 Mbps TX rate but relatively high current consumption of ~300 mA^39^. Alternatively, the long-range modem (LoRa) provides a few kbps data rate with three-kilometer link budgets for current consumption of ~120 mA^40^. However, Bluetooth low energy (BLE) offers the best compromise between a data rate of ~Mbps and low current consumption of ~10 mA^41^. Due to the high presence of BLE mobile devices in urban areas, this solution was preferred as the wireless form of engagement with the platform. A simplified schematic of the proposed embedded system is shown in Fig. 1a.

This platform uses a current-mode readout, a widely used technique for acquiring signals from resistive nanosensors, fabricated using silicon nanowires^42^ and CNTs^43^. Depending on the sensor and its application, the readout interface must be compatible with current values ranging from pA to µA. For instance, the CNT has a typical resistance of ~100 kΩ to 20 MΩ^44,45^, resulting in a current from 1 µA to 5 nA (bias = 100 mV). For this purpose, the embedded platform features integrated circuits capable of acquiring such low currents by a current to digital converter (CDC) and applying a potential bias with the help of a Digital to Analog Converter (DAC) to nanosensors.

## Sensor bias block (SBB)

An adjustable, reprogrammable bias is highly desirable during nanosensor operation. As illustrated in Fig. 1b, the potential bias of the sensor is software-defined and converted by a 12-bit DAC MCP4922^46^, offering 1.25 mV resolution on each channel. The software-based solution allows for easy adjustment of measurement conditions and parameters, such as sensitivity or current baseline^47,48^, which can be dynamically tuned over time, and extendable towards advanced, automated calibration procedures if desired. For a single 5 V battery-operated platform, an additional negative voltage is locally generated and doubled by using two charge pumps MAX660^49^ connected in cascade, as presented in the bottom part of Fig. 1b. The latter allows the potential bias to be programmed in the [−10 V… + 5 V] range with a 3.65 mV resolution. In Supplementary Section 1, Eq. (1), the derivation is provided.

## Sensor signal acquisition (SSA)

A multichannel CDC is desirable for acquiring and digitizing the nanosensor currents. Figure 1c shows the detailed schematic of DDC114^50^ time-interleaved integrators in “Convert Configuration” and “Integrate Configuration” with the timing diagram. The front-end integrators are followed by dedicated ADCs (16 or 20-bit configurable resolution) connected to the serial output interface. This solution offers true integration with a variable sampling rate and a Full Scale (FS) range programmable by two parameters: *T*_conv_ and *C*_range_, the integration time, and integrator capacitance. The timing of the CDC is critical for accurate operation, thereby influencing the high precision results. For this purpose, an external interruption timer integrated into the µC ensures accurate clocking of the CDC integrators. A variable sampling rate ranging from [0.001…3.125] kSPS has been achieved by programming the *T*_conv_ period in the [2000…0.64] ms range interval.

The *C*_range_ is set by a combination of three dedicated digital signals, which select one out of eight possible values formed by the CDC integrated capacitor bank of [3, 12.5, 25, 50] pF^50^. The resulting CDC FS output equation is presented in Supplementary Section 1, Eq. (2). Those two CDC parameters allow the system to dynamically configure its FS current range from 1.5 nA to 7.2 µA. In addition, this solution allows daisy chain connection possibilities, thereby facilitating the data shift through multiple devices. Consequently, the control signals are shared to maintain minimal digital control overhead^50^.

**Fig. 2:**
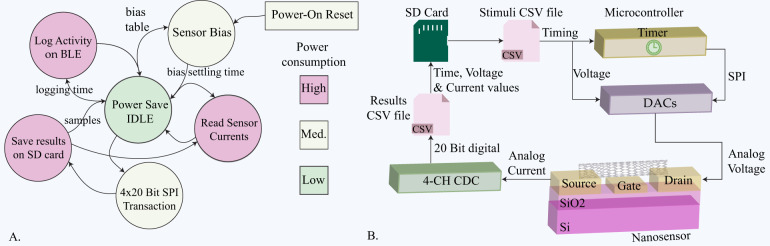
**a** Event-triggered Finite State Machine^60^, the internal states of the FSM are represented within the circles. The arrows represent the transition between individual states, with the logic condition annotated if applicable. The power consumption of each state is highlighted by the corresponding color of Low, Medium, and High power levels. **b** SD file system and block configuration illustrating the Stimuli.CSV file feeding the µC timers configuring internal interruption for FSM execution and external interruptions for the CDC together with the desired bias voltages for the DACs. Consequently, this timing and voltage amplitude is applied to the nanosensor terminals, and the analog currents are digitized by the CDC and saved on the SD card as Results.CSV file

An event-triggered finite state machine (FSM) operating on the µC has been realized for sensing routine automation. Each of the states and transitions presented in Fig. 2a is defined to perform a single discrete action, such as programming the bias voltage amplitude and duration, controlling the CDC configuration, storing measurements on the SD card, or transmitting the data via BLE. The state transitions of the FSM can be reconfigured with the help of a comma-separated file (CSV) stored on the SD card. The CSV file contains a customizable potential bias scheme that operates the nanosensors for a predefined time interval. The resulting current measurements are stored in a separate CSV file on the SD card. An overview of the configuration file system is shown in Fig. 2b.

## Results

### Wireless platform characterization

**Fig. 3:**
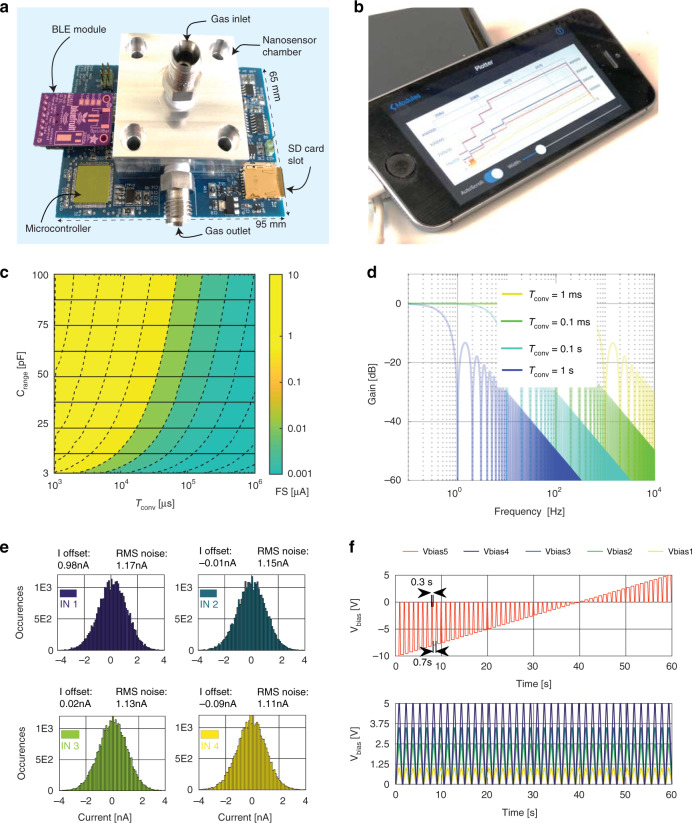
**a** An image of the embedded platform with highlights of primary building blocks, including a test chamber in the middle with the gas inlet on the top and gas outlet is on the side. The BLE module is on a separated breakout board attached to the platform. **b** Smartphone connected over BLE in advertising mode showing bias levels of the four individual sensors. **c** The CDC FS Range was obtained by tuning the two configuration parameters *C*_range_ and *T*_conv_ individually. **d** The resulting bandwidth of front-end discrete-time integrators after configuring a *T*_conv_ time interval. **e** The resulting input offset and RMS noise of the CDC including the parasitic contribution of the PCB with the four inputs in an open state. **f** An example of the bias block for the unipolar *V*_bias1_ to *V*_bias4_ (bottom) and the bipolar *V*_bias5_ (top) output including a timing example of one second bias period wherein the duty cycle is 0.7 s ON and 0.3 s OFF

The platform was designed to accommodate a sealed test chamber with a gas inlet and outlet on the PCB (see Fig. 3a). This allows for a controlled gas exposure of nanosensors under lab conditions. A smartphone paired with the platform via BLE shows tests current signals as illustrated in Fig. 3b. The SSA and the SBB are among the most critical parts of the signal acquisition path. The platform’s FS represents the maximum input current value (common for all channels, IN1 to IN4) of the SSA and is determined by the CDC’s two *T*_conv_ and *C*_range_ programmable parameters. The FS range is presented in Fig. 3c by the corresponding level contours. The platform’s bandwidth (BW) is given by the front-end integrators of the CDC^50^. They operate as classical continuous-time integrators wherein the feedback capacitor *C*_range_ accumulates charge for a predefined integration time *T*_conv_. Their derived transfer function can be found in Supplementary Section 1, Eq. (3). To fine-tune the SSA frequency response, one can set a *T*_conv_ parameter as shown in Fig. 3d. Various features of the platform, such as the noise, parasitic capacitances, and leakage currents originating from the PCB tracks, socket, and ceramic package, have been evaluated. With the four channels in an “open” state, Fig. 3e shows the input-referred current RMS_noise_ together with the current offset. A test bias file has been used for characterizing the SBB, as illustrated in Fig. 3f, where the five *V*_bias_ programmed in a staircase voltage step are shown. The values and the shape are adjustable with a predefined time step using the Stimuli.CSV stored on the SD card.

### NO_2_ sensing using a CNT nanosensor

For the SoA demonstration of the sensing platform, we refer to a CNT device (transfer and output characteristics presented in Supplementary Fig. S2) as a resistive nanosensor, exposed to NO_2_ gas. The suspended architecture and the residue-free fabrication process flow of the CNT device are detailed in ref. ^51^. A short description is presented in Supplementary Fig. S3. Aspects of the general gas sensor key performance parameters are presented elsewhere^35,48,52^. Measurements of the CNT nanosensor were performed at atmospheric pressure by using a customized gas mixing setup. A detailed description of the setup can be found in ref. ^53^. The CNT nanosensor was exposed to NO_2_ gas concentrations of [0, 200, 150, 100, 50, 10, 1, 0, 0] ppb under constant dry airflow^54^. The concentration steps were chosen to start from high to low NO_2_ values preceded by dry air exposure for two main reasons: first, to define a baseline of the CNT nanosensor drain current in the absence of NO_2_ gas, and second, to highlight the effectiveness of CNT nanosensor reset by evaluating this baseline. Experimental evaluation of the baseline concerning sensing bias voltage and reset time/energy is presented in Supplementary Figs. S4 and S5. For the current set of experiments, the FSM has been programmed to acquire consecutive samples with a temporal delay of 1/3 s in between. Denoted as *τ* in ref. ^55^, this sampling period has been chosen due to strong signal correlation, given by the 1/*f* noise, and mitigating the white noise with the LPF effect. For the same CNT devices, the influence of the observation window and the sampling frequency has been investigated in a previous work which can be found in ref. ^55^. Depending on the application requirements, the sampling rate of the embedded platform can be increased up to 3.125 kSPS which offers sufficient BW for acquiring a large variety of bio-signals^56^. The detailed sampling structure and the sampling rate power consumption overhead are presented in Supplementary Figs. S6 and S7.

**Fig. 4:**
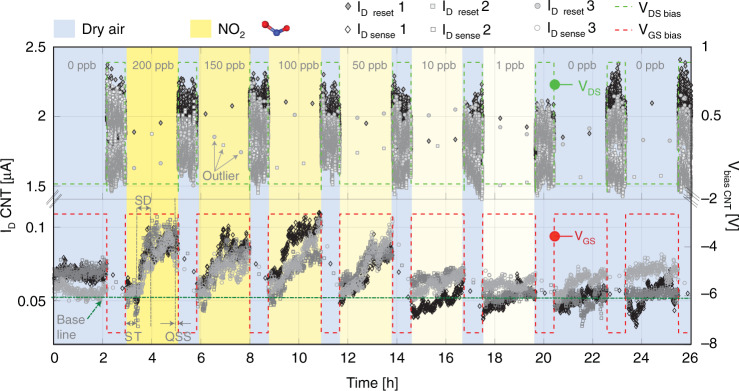
Three superimposed data sets with raw CNT current measurement (the filled points represent reset current samples and the unfilled points represent sense current samples) of the same experimental design, i.e., exposure to a decreasing NO_2_ gas concentration from 200 to 0 ppb. For each of the experiments, the settling time (ST) of the gas setup is taken into account, in addition, the bias level, baseline, and time windows for slope detection (SD) and quasi-steady-state (QSS) of the CNT nanosensor are highlighted. Bias in sensing regime is at *V*_GS_ = −2.7 V and *V*_DS_ = 0.1 V; bias in reset regime is at *V*_GS_ = 7.5 V and *V*_DS_ = 0.9 V

The experiments in Fig. 4 present a reproducible, current response of the CNT nanosensor to NO_2_ exposure. For the CNT nanosensor reset, a self-heating (SH) operation was performed after each concentration of NO_2_ exposure. The SH effect enables an accelerated gas desorption mechanism, as observable in the top part of Fig. 4.

In this bias region, the CNT current is saturated, which induces the SH onset resulting in a negative-differential conductance behavior^45^. The bottom part of Fig. 4 shows the CNT drain current values when exposed to NO_2_, biased at a *V*_GS_ = −2.7 V and *V*_DS_ = 0.1 V. In addition, the top part of Fig. 4 shows the sensor recovery window at an elevated bias voltage of *V*_GS_ = −7.5 V and *V*_DS_ = 0.9 V after each exposure sequence (experimental determination of these bias levels are presented in Supplementary Figs. S4, S5, and S8). The current samples denoted as “outlier” in Fig. 4 can be ignored since they represent CDC’s first integration cycle^50^ immediately after power-on-reset. The experimental sequence was repeated thrice at the same bias levels and NO_2_ gas concentrations for consistency. Significant repeatability and the effective sensor reset between the measurements data sets [#1, #2, #3] (gray level) are observable in Fig. 4.

## Discussion

### CNT nanosensor signal evaluation

One of the widely used measures to characterize the sensing performance of a transducer is the LOD value. This performance parameter is represented by the lowest NO_2_ concentration for the CNT nanosensor, measured with a three-sigma (3*σ*) confidence interval. Compared to another type of sensor response (i.e., AlphaSense^57^ response presented in Fig. S9), the signal evaluation of the current CNT device is based on the former research work^55^ of the group, which presents an extensive analysis of slope detection (SD) versus quasi-steady-state (QSS) sensing regimes. By observing the CNT nanosensor current evolution over time, three different regions within a NO_2_ exposure pulse are highlighted in Fig. 4. The regions are named as (i) settling time (ST) of ~20 min, (ii) SD region from five to 20 min, and (iii) QSS during the last 5 min. Langmuir isotherm model^58^ can be used to analyze the adsorption state on the CNT nanosensor surface. According to this model, the initial slope of the current signal dependency upon gas concentration can be expressed as $d\theta \left( {t = 0} \right)/dt = K_{\rm{ads}} \cdot p$, wherein *θ* represents the CNT nanosensor surface coverage, *p* is the analyte concentration or partial pressure and $K_{\rm{ads}}$ is the adsorption coefficient. This shows the advantage of the initial slope signal, which is linearly proportional to the gas concentration under evaluation. An ST of 20 min was considered after the NO_2_ gas flow was started, as depicted in Fig. 4. After the ST, the initial slope, SD response of the nanosensor is investigated at various time windows ranging from five up to 20 min.

**Fig. 5:**
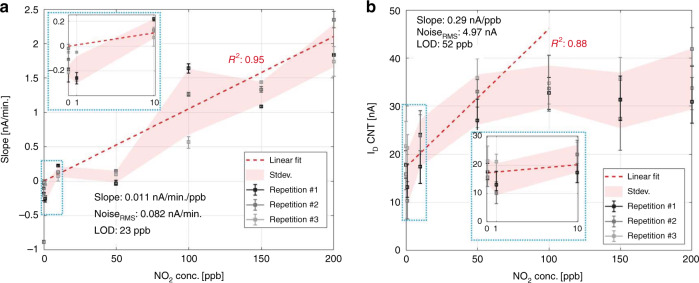
**a** The current slope of the first 12-min transient CNT nanosensor response. Inset: Magnified transient response (blue-dotted square) of the nanosensor from 0 to 10 ppb. **b** The CNT nanosensor’s last five-minute quasi-steady-state (QSS) response when exposed to NO_2_ gas concentrations Inset: Magnified QSS (blue-dotted square) of the nanosensor from 0 to 10 ppb. The error bar length highlights the total electronic noise (i.e., the noise of the CNT nanosensor and the embedded platform) and the NO_2_ target concentration inaccuracy over exposure time. The spread between the error bars at a given gas concentration represents the CNT nanosensor response variation, including relative inaccuracy of the gas setup. Note: Each data point represents an average and the standard deviation of 60 samples acquired at fixed bias conditions common for (**a**) and (**b**): *V*_DS_ = 0.1 V and *V*_GS_ = −1 V

In Fig. 5a, the initial slope of the CNT nanosensor current response during the first 12 min of NO_2_ exposure in the SD region is presented. Excellent sensor linearity can be observed within this time window, evaluated using the linear fit coefficient of determination *R*^2^. Estimation of the LOD and *R*^2^ vs. the time window size is detailed in Supplementary Fig. S10.

The result presented in Fig. 5b shows the data from Fig. 4 denoted as QSS, wherein the average steady-state current response during the last 5 min of the 2-h NO_2_ exposure is evaluated as CNT nanosensor sensing response. Using the linear fit shown in Fig. 5a, b as the device calibration curve and including the resulting error bars as being the noise of the three acquired samples, the LOD can be determined by 3*σ*·root-mean-square (RMS) noise divided by the gas response slope at low gas concentrations.

Here, the noise_RMS_ is calculated as the RMS value of the slope’s standard deviation across individual current signal response samples at [10, 1, 0] ppb NO_2_ concentration. The shaded area of Fig. 5 illustrates the standard deviation around the average current value for all measurement data sets [#1, #2, #3] at each gas concentration. Using the data and their respective fits as shown in Fig. 5a, the LOD limit values are calculated as in

1 $${{LOD}_{{SD}}} = 3 \cdot \frac{{noise}_{{RMS}}}{{slope}} = 23.4\,{{ppb}} \approx 23\,{{ppb}}$$

And from Fig. 5b where the QSS sensing regime is explored

2 $${{LOD}_{{QSS}}} = 3 \cdot \frac{{noise}_{{RMS}}}{{slope}} = 51.9\,{{ppb}} \approx 52\,{{ppb}}$$

Operating CNT nanosensor by pulsed SH and SD, concentrations of NO_2_ below 23 ppb can be resolved. It has been highlighted that the initial slope sensing based on SD can dramatically decrease the response time, offering both better linearity and dynamic range (see: Fig. 5a). In Fig. 5b, the classical approach of SS or QSS is explored, wherein the Langmuir isotherm flattening is observable at higher gas concentrations due to the complete surface coverage^58^. In addition, the CNT nanosensors fabricated using the ultra-clean, dry-transfer technique show a significant reduction in sensitivity to humidity^59^. The humidity cross-sensitivity experimental result of the CNT nanosensor is presented in Supplementary Fig. S11.

### Embedded platform power consumption

**Fig. 6:**
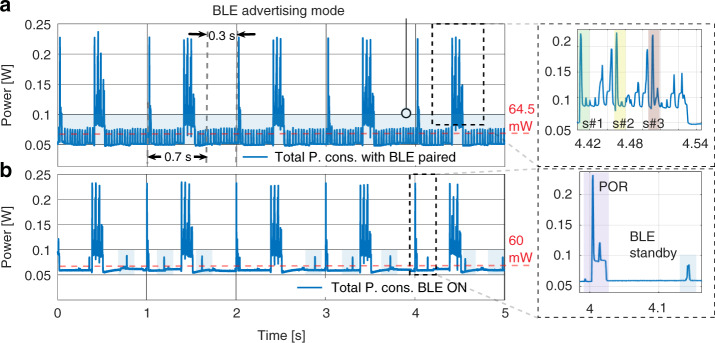
**a** Power consumption of the embedded platform while acquiring three SPS annotated as s#1, s#2, and s#3 wherein the BLE power consumption is visible in the continuous peaks when operating in advertising mode compared with **b** wherein fewer power peaks are observed when BLE is ON but not paired. The POR (power-on reset) power consumption is also visible when the IDLE state is left at each new bias period of one second with the corresponding duty cycle

The embedded platform has been supplied by a 5 V; 2800 mAh battery, and the power consumption has been monitored during the operational states. An IDLE state was defined to switch off unnecessary peripherals and execute µC power-save mode. According to the Stimuli.CSV file, a single timer is kept operational in this state, responsible for waking the remaining peripherals according to the Stimuli.CSV file. Figure 6 showed the platform’s power consumption when three current response results in a row were acquired every second, including the IDLE state in-between. This sampling rate corresponds to the typical energy consumption of an environmental monitoring station sampling at three SPS denoted as [s#1, s#2, s#3]. A low sampling rate of three SPS is preferred in this particular application for lowering the power consumption but still being fast enough in collecting sufficient samples and achieving slope-detection within a 5-min observation window for a (3*σ*) LOD of ~90 ppb (as illustrated in the Supplementary Fig. S10).

In Fig. 6a, the average power consumption of 64.5 mW can be observed when the proposed platform executes the custom FSM states of Fig. 2a, and the BLE is paired in advertising mode at 0 dBm TX power. The peak power consumption of about 225 mW corresponds to the CDC acquisition and SD card data storage. In Fig. 6b, the average power consumption drops to 60 mW when the BLE is ON but not paired with a mobile device. The average power consumption values are determined not considering computing power for signal evaluation. A comparison with the theoretical power consumption for the platform’s main components can be found in the supplementary Table S1.

However, the energy efficiency of the proposed platform can be further optimized by reducing, reordering, or customizing the software-defined FSM states and states transition timing. In comparison, a commercial reference platform, e.g., Aeroqual, which uses an SM-50 O_3_ measurement unit^11^ for outdoor environments, provides highly accurate ozone measurements within [0…150] ppb. However, it operates at a high minimum power consumption of 2.5 W, excluding wireless communication^11^. The Telaire 6713 from Amphenol Advanced Sensors, a sensor measuring indoor CO_2_ concentrations within [400…5000] ppb with high accuracy, suffers from a similar shortcoming^12^. While the sensor itself is suitable for wearables due to its form factor of 30 × 15.6 mm, its average power consumption of 135 mW without sensor electronics is relatively high for a long-term battery-operated system. A recently published full system solution is the W-Air module presented in^11^ employs two MOX for O_3_ and CO_2_ sensors from the shelf trying to eliminate the interference of VOC emissions. At a sampling rate similar to the one presented in this work, the system in^13^ draws an average power of 150 mW, twice the value compared to the average value presented in Fig. 6. The presented work confirms the preliminary results from^55^ by exploring sensing solutions with repetitive experiments and portable-embedded platforms at a fraction of total power consumption compared to lab equipment. A summary of the performance of the embedded system and the CNT nanosensor in comparison to selected gas sensing solutions is presented in Table 1.

## Conclusion

We presented the concept, realization, and performance evaluation of a portable, customizable embedded platform for nanosensor applications. The platform’s hardware can adapt to the demands of the nanosensor requirements and can measure a wide current range. In addition, our solution is fully autonomous and reconfigurable, employing a user-defined instruction set. The FSM’s embedded functions allow for setting various platform parameters, namely: the CDC integration time and capacitor bank, defining the FS and BW, DAC bias level/period (including a bipolar potential beyond the supply voltage), time intervals for SD card storage and BLE data transmission. Moreover, an additional power-saving FSM-state deactivates the µC’s internal blocks and thus reduces the average power consumption to 60 mW. The power bank can ensure up to nine days of continuous operation for the measurement protocol in this configuration. An application of the embedded platform has been demonstrated by integrating an ultra-sensitive CNT nanosensor. A reproducible CNT nanosensor response to NO_2_ exposure was demonstrated down to 1 ppb of NO_2_ in dry air with a 3*σ* LOD as low as 23 ppb (1*σ*: 7 ppb). Our customizable, compact embedded sensor platform demonstrates the unique capability of CNT nanosensor readout and enables validation of the respective annual exposure limits set by the EU. The user-defined software-based solution allows for simple addition, replacement, and reordering of FSM states, thus offering a high degree of flexibility and enabling further trade-off between functionality and energy efficiency.

## Supplementary information

Supplemetary Material
