# Supplementary material for: A customizable, low-power, wireless, embedded sensing platform for resistive nanoscale sensors
Source: Microsyst Nanoeng. 2022 Jan 14;8:10. doi: 10.1038/s41378-021-00343-1 (PMC8760339; doi:10.1038/s41378-021-00343-1)
Supplement: Supplementary file 1 — Supplemetary Material [file 41378_2021_343_MOESM1_ESM.pdf]

A Customizable, Low-Power, Wireless, Embedded Sensing Platform for Resistive Nanoscale Sensors

Stefan Nedelcu[1)], Kishan Thodkar, and Christofer Hierold

Micro- and Nanosystems, Department of Mechanical and Process Engineering, ETH Zurich,
Tannenstrasse 3, 8092 Zurich, Switzerland

Correspondence: [1)] stefan.nedelcu@micronano.ethz.ch tel./fax: +41 (0) 44 632 87 14

1. Sensor Bias Block and Signal Acquisition

Using the superposition principle applied on the linear op amp configuration, the V_{bias5} potential can be programmed in the $[-2V_{bat.} \dots V_{bat.}]$ range with a 3.65 mV resolution as:

$$V_{bias5} = V_{bias5}\Big|_{V_{DAC}=0} + V_{bias5}\Big|_{V_{bat.}=0} = 3 \cdot V_{DAC} - 2 \cdot V_{bat.} = \left(3 \cdot \frac{\#code}{2^{\#RES}} - 2\right) \cdot V_{bat.} \in [-2 \cdot V_{bat.}, V_{bat.}]\,. \quad (1)$$

where $V_{bat.}$ = 5 V is the battery voltage supply, $\#\mathbf{Res}$=12 bits is the DAC resolution and $\#\boldsymbol{code}$ the input code respectively**.**

By choosing the integration period $\mathbf{T_{conv}}$ and integration capacitance $\mathbf{C_{range}}$, the FS is programmable in the range of [1.5 $\mathbf{nA}$ … 7.2 $\mathbf{\mu A}$]. The output data format can be selected with a desired resolution, $\#\mathbf{RES}$,of [16; 20] $\mathbf{bits}$ which corresponds to an $\mathbf{I_{in\,LSB}}$of [1.4; 23] $\mathbf{fA}$ and [6.8; 109] $\mathbf{pA}$, respectively. The digital output can be expressed as:

$$\#Out_{FS}[1:4] = \left[\frac{I_{in[1:4]}[nA] \cdot T_{conv}[\mu s]}{C_{range}[pF] \cdot V_{REF}[mV]} \cdot (2^{\#RES} - 1)\right] + 2^{\#RES-8}\,, \quad (2)$$

where $V_{REF} = 4096$ mV is the DDC114’s voltage reference and the factor $2^{\#RES-8}$ depicts the read value at zero current input. This value corresponds to approx. 0.4% of the FS range [1] and represents a small offset intentionally introduced as a safety margin that prevents negative input currents due to PCB parasitics or leakage currents. The bandwidth (BW) is given by the front-end integrators of the DDC114 [1]. They operate as classical continuous-time integrators where the feedback capacitor C_{range} stores charge for the predefined integration time T_{conv}. The frequency response of the ADC that follows the front-end integrators doesn’t influence the BW [1] since it operates at a constant and higher sampling frequency.

Hence, the transfer function can be written as:

$$H(f) = \frac{1}{C_{range}} \cdot \frac{\sin(\pi \cdot T_{conv} \cdot f)}{\pi \cdot f} \qquad (3)$$

2. Example of NABEL measurements of NO2 average gas concentration

The practical relevance of the proposed platform is highlighted by the example of the daily NO_2 average value acquired by NABEL station [2] for two of Switzerland's biggest cities: Zurich and Lugano as presented in Figure S1.

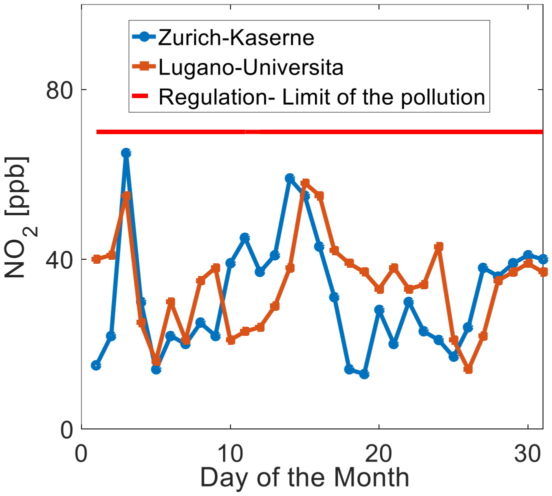

Figure S1. NO_2 air pollution measured in 2017; data reproduced from the published yearly report of NABEL station in Switzerland [2].

3. CNT characterization

A pre-characterization of the CNT nanosensor is performed before exposure to NO_2 gas analyte. In Figure S2a. the transfer characteristic of KTDS15, mounted in the test chamber under atmospheric conditions, is presented at different V_{DS} bias levels. The CNT nanosensor shows no pronounced–hysteresis, which is expected for suspended CNT device architecture [3]. The output characteristics of the CNT nanosensor is presented in Figure S2b, wherein the current values are acquired at different V_{GS} bias levels. The current curves demonstrate the different operational regions of the CNT nanosensor: 1) the linear region where the CNT device current is linearly proportional to the applied bias and 2) the CNT device self-heating region which can be observed for elevated bias levels.

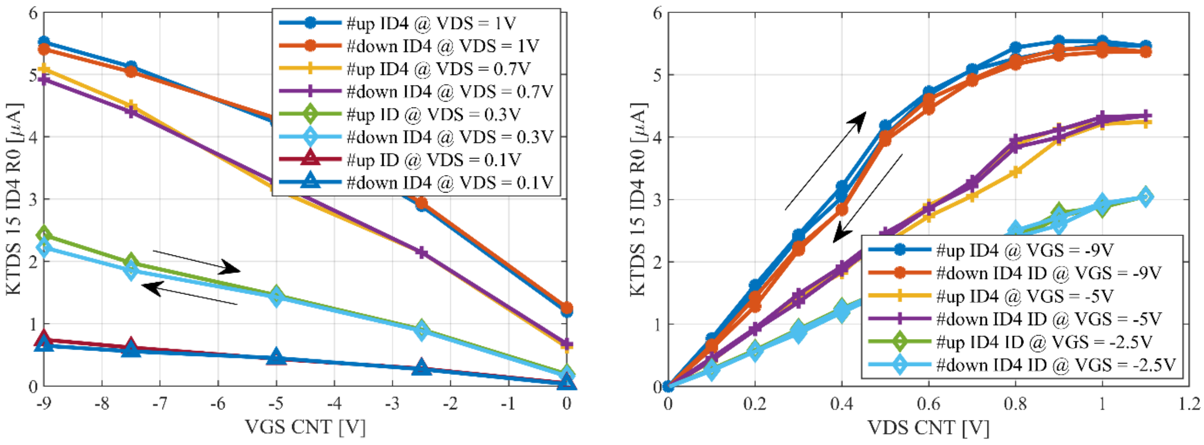

(a) (b)

Figure S2. (a) Transfer characteristics of the CNT device at different V_{DS} bias showing no substantial hysteresis; (b) Output characteristics of the same device at different V_{GS} bias showing the linear region and self-heating effect at elevated bias level. Note: ID4 refers to channel 4 and R0 refers to experiment number one.

4. CNT nanosensor fabrication

The architecture and process flow of the CNT devices is based on suspended CNTs as described recently in [4]. The as grown CNTs are mechanically transferred to the device substrate, comprising of mesa like Source (S) and Drain (D) electrodes allowing to assemble the CNTs suspended from the substrate and from the Gate (G) (see Figure S3a). The source and drain contacts are metallized before CNT transfer using thermal evaporation of Cr (1 nm) and Pd (40 nm). The drain current I_D is controlled by the gate, located under the suspended structure as shown in Figure S3Figure S3a. The CNT together with the substrate forms a suspended structure with a channel length of 2.8 µm and a gate distance of 1 µm. The suspended CNT architecture is advantageous in both sensing due to the increased surface area for adsorption as well as for increased SNR and decreased hysteresis [5] due to the absence of dielectric materials acting as charge traps. The detailed substrate fabrication process including CNT growth and transfer is presented in [4]. A top-view Scanning Electron Microscopy (SEM) image of a typical CNT device is shown in Figure S3b.

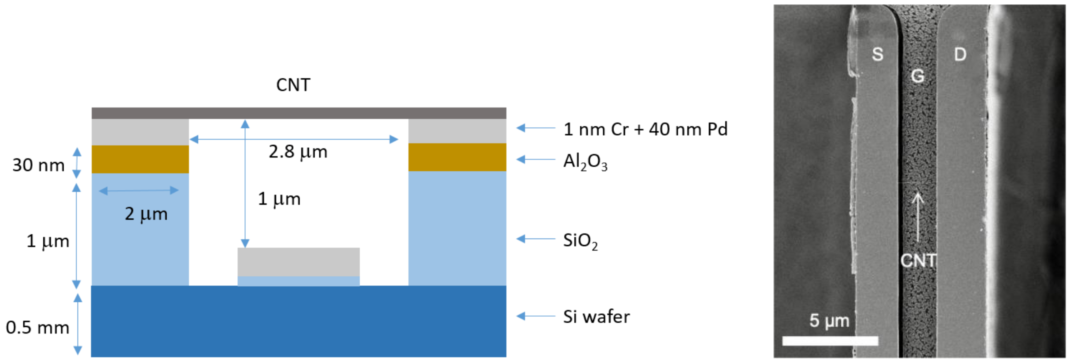

(a) (b)

Figure S3. (a) CNT-FET substrate illustration; (b) Top view SEM image of a suspended CNT (marked by the arrow).

5. **Experimental determination of bias voltages for CNT devices sensing and reset**

The measurement baseline is defined as being the nanosensor current level after a reset phase by the help of elevated bias voltage, which leads in the self-heating of the CNT device as Joule a thermal effect. During the set of experiments presented in the manuscript, the CNT device has been exposed to a gradually decreasing NO_2 gas concentration followed by a reset state. One can quantize the reset efficiency by evaluating the offset of the baseline current after each reset phase. By exposing the CNT device from high to low NO_2 gas concentration one can observe a proper reset phase given by a straight baseline. This means that the reset phase duration and bias levels are properly tuned to reach the same baseline current after both high and low NO_2 gas exposure. For example, a long reset phase would lead in a baseline current undershoot after a low gas concentration exposure. In contrast, short reset phase would lead in an incomplete desorption after a short reset phase at a high gas concentration exposure. This can be highlighted by two additional experiments presented in Figure R5 and Figure R6, which compares the resulting currents levels immediately after the reset and exposure phase at different bias levels and reset time intervals. In Figure R5, at a sensor bias of 100-200 mV, an optimal sensor response is observable. Furthermore, at a Self-Heating time period of 45 minutes we can observe optimal sensor reset (see: Figure R6). These parameters were utilized for performing the measurement in Figure 4.

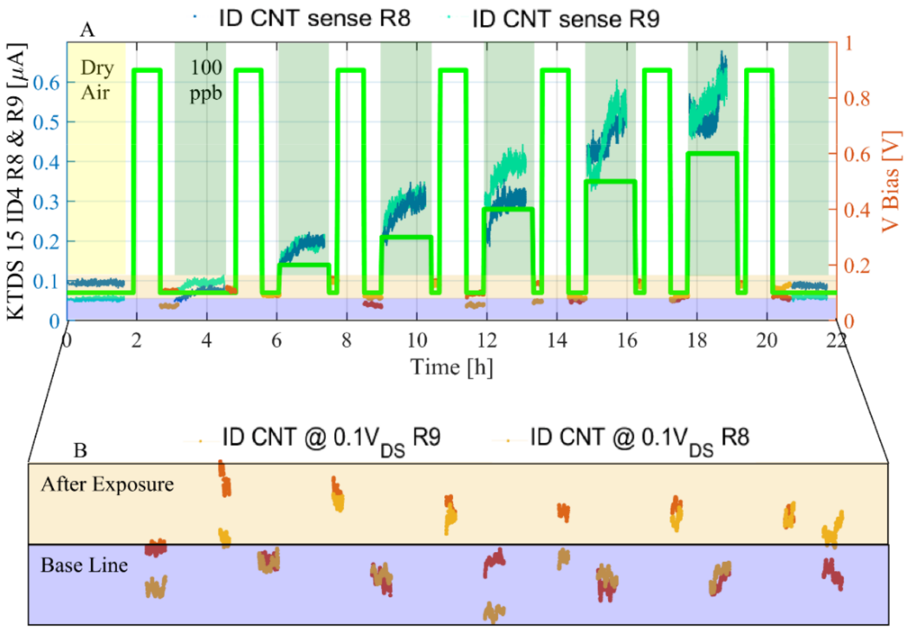

Figure S4 A) CNT sensor signal response at 100 ppb NO_2, constant V_{GS} and variable V_{DS}. B) Magnification of the baseline current levels after reset and after NO_2 gas exposure.

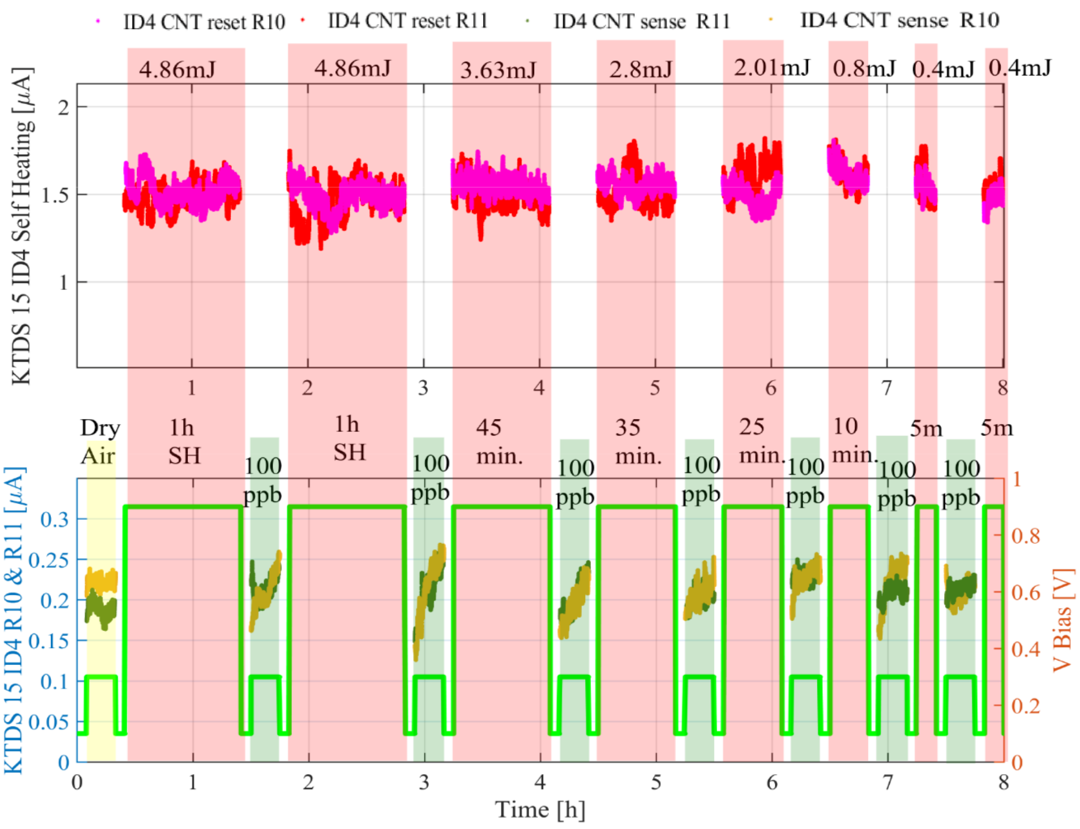

Figure S5 (top) CNT reset current levels and the corresponding desorption energy (bottom) CNT nanosensor signal response at 100 ppb NO_2, constant V_{GS}; V_{DS} and variable reset time.

6. Sampling Frequency Power Consumption Overhead

Each sample of the 3 SPS in the current implementation is composed of 32 averaged samples, with 4 samples intentionally discarded during the first integration cycle. Figure S6 shows the detailed capture of the DDC114 sampling signal. Note: each edge represents one sample [1].

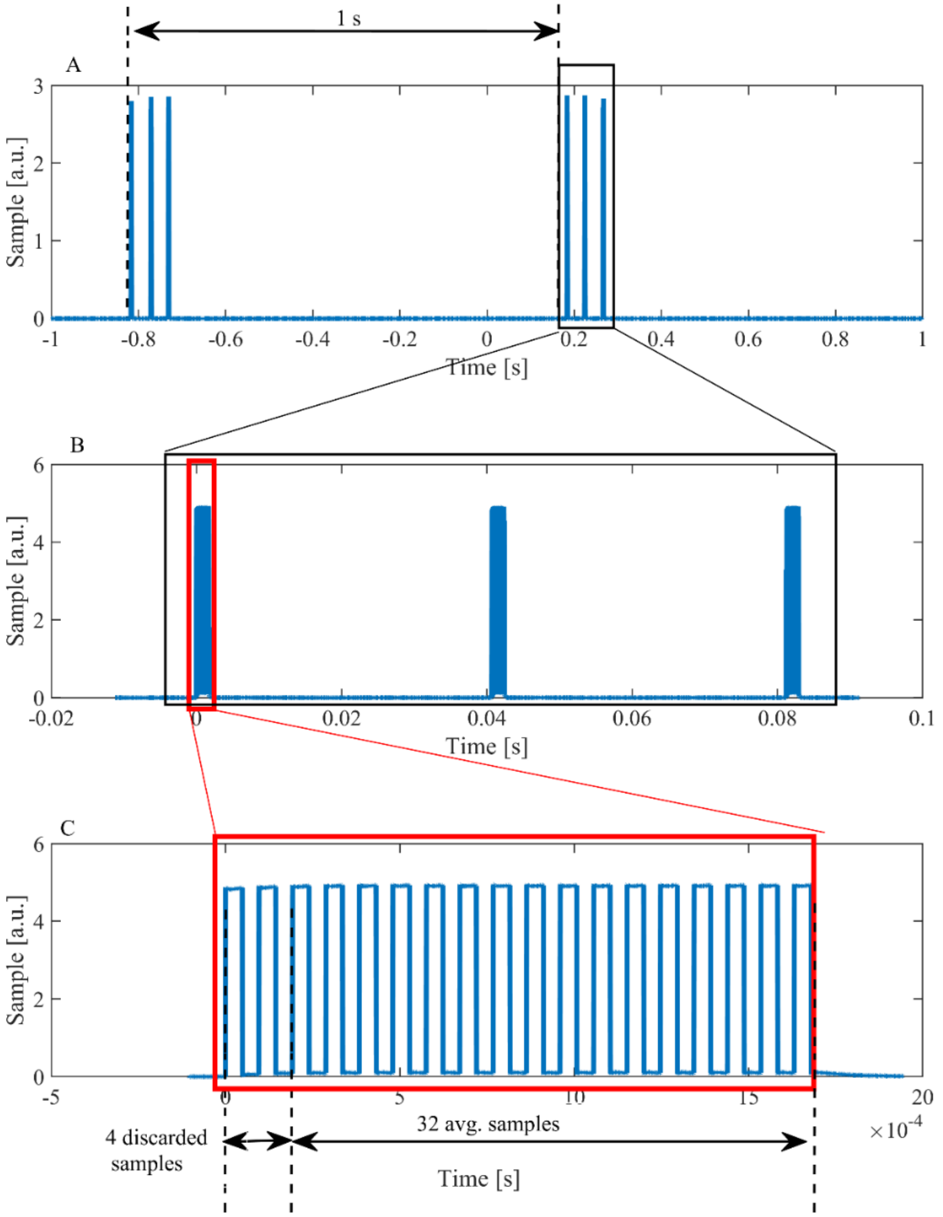

Figure S6 A) The current sampling scheme for the DDC114 resulting in 3 SPS programmed by the FSM. B) The detailed sampling structure of the 3 SPS burst signal. C) The zoom-in signal structure of an individual sample presented in detail.

In this case, the DDC114 power consumption overhead is not of concern. Measurement results of the DDC114 at different sampling rates are presented in Figure S7.

A.

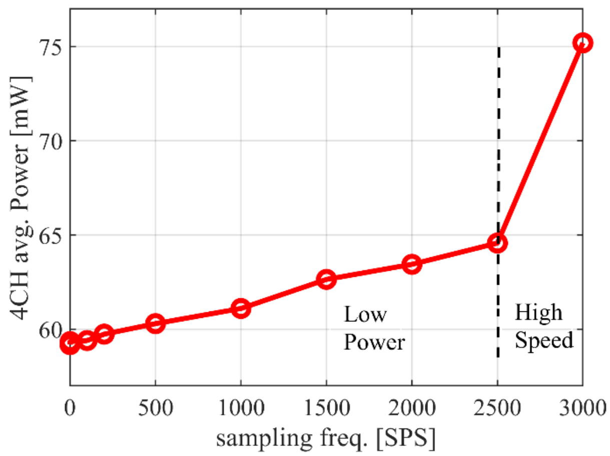

B.

Table 4. HISPD/LOPWR Pin Operation

HISPD/LOPWR	MODE	TYPICAL POWER/ CHANNEL	MAXIMUM CLK FREQUENCY (CLK_4X = 0)	MAXIMUM DATA RATE
0	Low Power	13.5mW/ch	4.0MHz	2.5kSPS
1	High Speed	18.0mW/ch	4.8MHz	3.125kSPS

Figure S7A) DDC114 power consumption versus the sampling frequency. B) Peak power consumption reported in the DDC114 datasheet [1].

The measured values are for all the four channels of the DDC114. Note: the measured sampling rate matches with the values reported in Table 4 of the datasheet [1]. The minor difference compared to B) is given by the analog voltage reference REF3140 [6], which is required for the DDC114 to operate.

8. Experimental determination of bias voltages for CNT devices sensing and reset

Thermal heating effects are amongst the prominent limitations due to the application of elevated voltage levels to the CNT nanosensor. Over extended period of time, these can lead i) to chemical oxidation in presence of O_2 and ii) an increased electronic noise due to the thermal agitation of charge carriers. An additional measurement set describing these effects is presented in Figure S8.

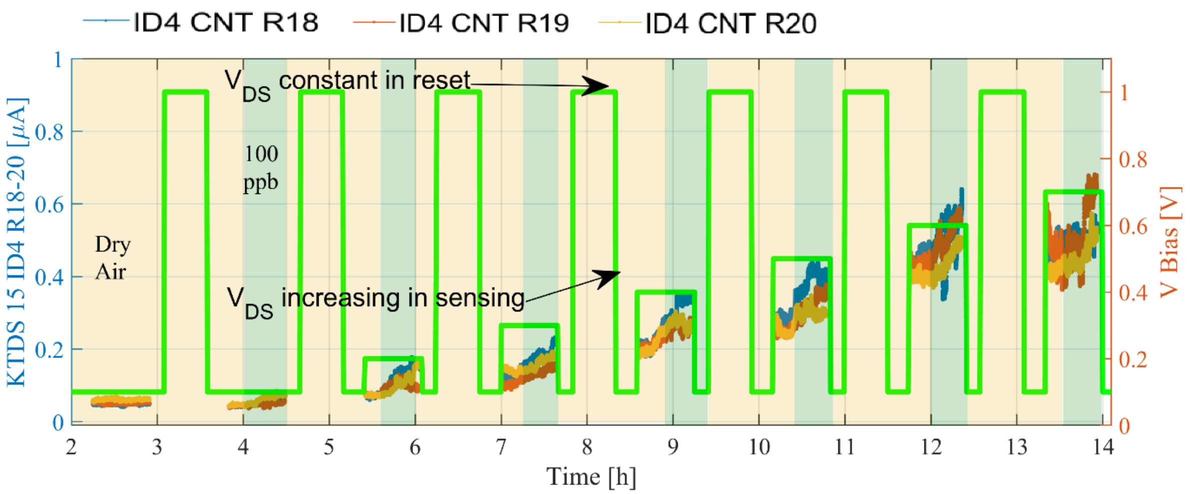

Figure S8 CNT sensor signal response at 100 ppb NO_2, constant V_{GS} and variable V_{DS}. The reset phase of the CNT device is not shown.

In the experimental data shown in Figure S8, the CNT nanosensor is successively exposed to a constant NO_2 gas concentration of 100 ppb after a preliminary DA (Dry Air) exposure. After a CNT nanosensor reset phase (not shown in this graph), the successive gas exposure is performed at a gradually increased V_{DS} in steps of 150 mV. It can be observed that the current signal value and it's corresponding slope increases with V_{DS} values up to V_{DS} = 450 mV. However, for the responses with V_{DS} > 450 mV, an increasing current is noticeable but the corresponding slope decreases. This could be explained by considering the thermal heating effects leading to the desorption of NO_2 molecules from the CNT nanosensor surface. As highlighted earlier, we can observe an increased noise level of the current signal at V_{DS} > 450 mV. This experiment has been repeated thrice for consistency.

9. AlphaSense signal response acquired by the proposed platform when exposed to NO_2 gas analyte

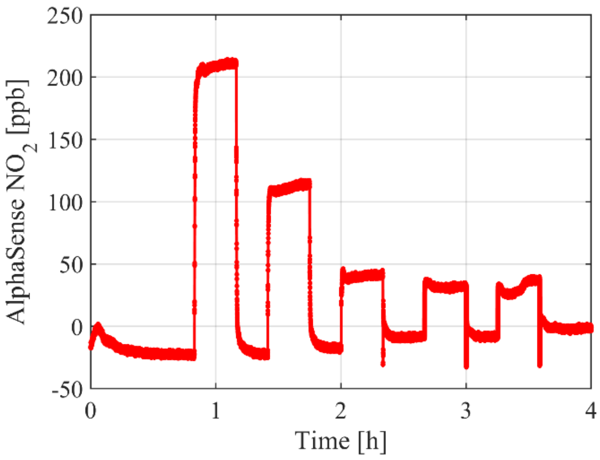

Figure S9 AlphaSense signal response acquired by DDC114 on exposure to NO_2.

10. Experimental determination of LOD and R^2

The reported response time of 12 minutes corresponds to the lowest LOD=23 ppb (3σ) and the highest linearity evaluated by the help of R^2 linear fit parameter. However, this can be decreased down to 5 minutes, as presented in Figure S10 with an LOD of ≈90 ppb (3σ). Herein still comparable to other sensing materials reported by the articles highlighted in Table 1 of the manuscript.

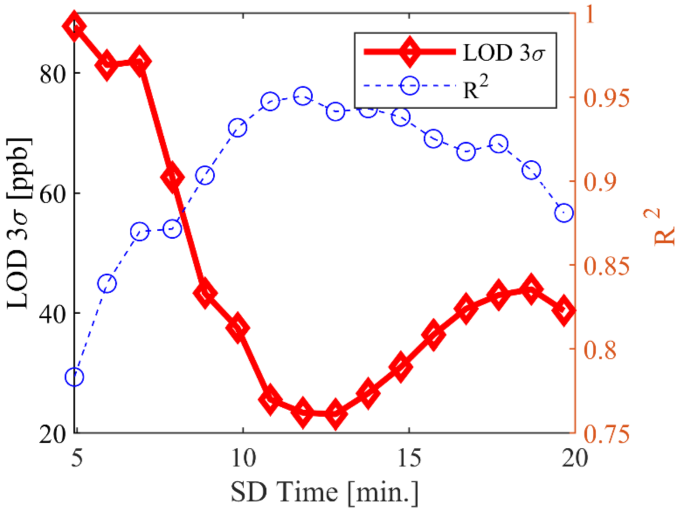

Figure S10 LOD (red filled diamond) and R^2 coefficient (blue circle) vs. Slope Detection (SD) time window.

Note: The SD time of 12 minutes was identified as good choice and applied in Figure 5A of the main text.

11. Humidity Cross-Sensitivity

In Figure S11, the influence of relative humidity pulses has been evaluated in comparison to dry air conditions. Reduced cross-sensitivity to humidity is observable for gas flow conditions with 0 and 100 ppb NO_2 gas concentration.

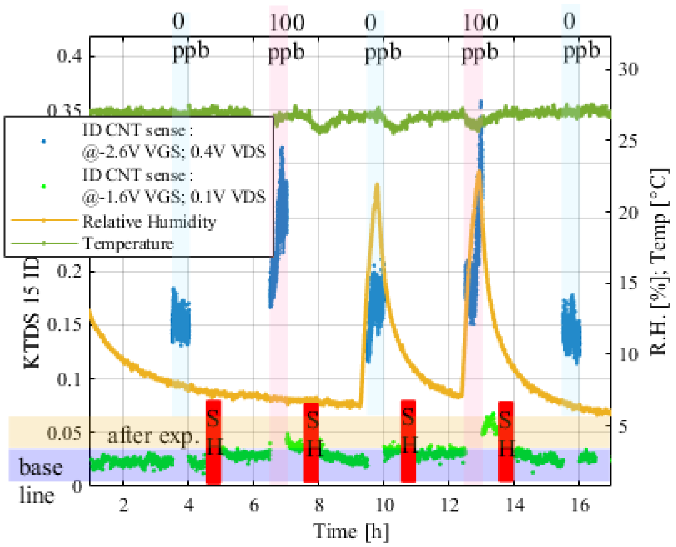

Figure S11 CNT nanosensor signal response at 0 and 100 ppb NO_2 gas concentration in the absence and presence of humidity

12. Power Consumption

The reported power consumption of main components in Active and IDLE mode is calculated as in Table S1.

Table S1

Device	Active	IDLE	REF
ATmega2560	13.2 mW	0.66 uW	[7]
SD card	200 mW	3.1 mW	[8]
DDC114	54 mW	-	[1]
REF DDC	412 uW	-	[9]
MAX 660	1.25 mW	-	[10]
MCP4922	4.3 mW	50 uW	[11]
nRF51 BLE	43 mW	5 mW	[12]

References

1. Texas Instruments Quad Current Input, 20-Bit Analog-To-Digital Converter Available online: https://www.ti.com/lit/ds/symlink/ddc114.pdf (accessed on Dec 1, 2020).
2. Federal Office of the Environment FOEN - Data query Available online: https://www.bafu.admin.ch/bafu/en/home/topics/air/state/data/data-query-nabel.exturl.html (accessed on Jan 14, 2021).
3. Chikkadi, K.; Muoth, M.; Maiwald, V.; Roman, C.; Hierold, C. Ultra-low power operation of self-heated, suspended carbon nanotube gas sensors. *Appl. Phys. Lett.* **2013**, *103*, 223109, doi:10.1063/1.4836415.
4. Jung, S.; Hauert, R.; Haluska, M.; Roman, C.; Hierold, C. Understanding and improving carbon nanotube-electrode contact in bottom-contacted nanotube gas sensors. *Sensors Actuators B Chem.* **2021**, *331*, 129406, doi:10.1016/j.snb.2020.129406.
5. Chikkadi, K.; Muoth, M.; Liu, W.; Maiwald, V.; Hierold, C. Enhanced signal-to-noise ratio in pristine, suspended carbon nanotube gas sensors. *Sensors Actuators B Chem.* **2014**, *196*, 682–690, doi:10.1016/j.snb.2014.02.058.
6. REF3140 Available online: https://www.ti.com/product/REF3140?keyMatch=REF3140.
7. 8-bit Atmel Microcontroller with 16/32/64KB In-System Programmable Flash.
8. TDK SD Card / Micro SD Card Product Specifications Available online: https://product.tdk.com/en/system/files?file=dam/doc/product/flash-storages/flash-storages/sd-card/catalog/flashstorage_sd-card_mmrd4_murd4_en.pdf.
9. Townsend, K. Introducing the Adafruit Bluefruit LE SPI Friend 2018, 81–82.
10. Integrated, M. MAX660 Switched Capacitor Voltage Converter Available online:

https://datasheets.maximintegrated.com/en/ds/MAX660.pdf (accessed on Dec 1, 2020).

11. Microchip MCP4922 12-Bit Dual Voltage Output Digital-to-Analog Converter with SPI Interface Available online: https://ww1.microchip.com/downloads/en/devicedoc/22250a.pdf (accessed on Dec 1, 2020).

12. nRF52805 Product Specification v1.2 Available online: https://infocenter.nordicsemi.com/pdf/nRF52805_PS_v1.2.pdf (accessed on Dec 1, 2020).
